# Identification of high-risk patients and metastatic progression in primary c/pN0 oral squamous cell carcinoma

**DOI:** 10.4317/medoral.28176

**Published:** 2026-04-19

**Authors:** Riikka Eloranta, Rayan Nikkilä, Suvi Tuuli Vilén, Tommy Wilkman, Tuula Salo, Johanna Snäll

**Affiliations:** 1 Department of Oral and Maxillofacial Diseases, Clinicum, Faculty of Medicine, University of Helsinki and Helsinki University Hospital, Helsinki, Finland; 2 Research Program in Systems Oncology, Faculty of Medicine, University of Helsinki, Helsinki, Finland; 3 Department of Oral and Maxillofacial Diseases, Kymenlaakso Central Hospital Kotka, Finland; 4 Translational Immunology Research Program (TRIMM), University of Helsinki, Helsinki, Finland; 5 Research Unit of Population Health, Faculty of Medicine, University of Oulu, Oulu, Finland; 6 Medical Research Center, Oulu University Hospital, University of Oulu, Oulu, Finland; 7 Department of Pathology, HUSLAB, Helsinki University Hospital, University of Helsinki, Helsinki, Finland

**Keywords:** Oral cancer, squamous cell cancer, metastasis, cervical lymph node, survival.

## Abstract

**Background:**

Metastasis significantly decreases survival in oral cavity squamous cell carcinoma (OSCC). Even patients initially staged as cN0 may develop cervical or distant metastases during follow-up. Risk factors contributing to metastasis in this group are not fully understood.

**Materials and Methods:**

A retrospective cohort study was conducted on patients with primary cN0 OSCC treated between 2016 and 2020.

**Results:**

Of the 195 N0 OSCC patients included, 19 (9.7%) developed metastasis. Heavy alcohol use (HR 4.05, 95% CI:1.38-11.92, p=0.011) and depth of invasion (DOI) (HR 1.10, 95% CI:1.01-1.20, p=0.021) were significantly associated with the development of metastasis. Age, gender, site, neck procedure, radiotherapy, and T-class were not statistically significant predictors in this analysis.

**Conclusions:**

Metastases occurred in nearly 10% of c/pN0 patients. Alcohol use and DOI were independent predictors, highlighting the need for individualized follow-up strategies and further research into alcohol-related OSCC.

## Introduction

Recurrence and metastasis occur in approximately 50% of patients with head and neck squamous cell carcinoma (HNSCC) within the first 2 years [1]. Oral cavity squamous cell carcinoma (OSCC) is primarily treated with surgery and the overall survival rate of the disease ranges from 33% to 70% depending largely on the disease stage, treatment modalities, and other disease-specific factors [2,3]. The presence of cervical lymph node metastasis (LNM) at the time of the diagnosis of OSCC is considered the most important prognostic factor since it can decrease the 5-year survival by 50% [4,5]. The 5-year survival rates for OSCC patients staged N0, N+ and M1 have been reported to be 86%, 69% and 40%, respectively [6]. The incidence of occult LNM in OSCC has been reported to range from 21% to 42% in previous studies and may be associated with the location of the primary tumor [7,8]. Still, despite advances in screening, imaging techniques, and treatment modalities, a considerable number of patients experience metastases [9,10]. The underlying reasons for the delayed onset of metastases in some patients remain unclear.

According to previous studies, 1-34% of pT1-T2N0 OSCC patients develop LNM within 2 years postoperatively [11,12]. In addition to variations in LNM rates, the mean time to diagnosis of regional recurrence also differs across studies from 6 to 14 months [13-15]. While differences in patient populations and study settings account for some of this variation, numerous other factors contribute to the outcomes. Factors associated with LNM development in OSCC include advanced T-stage [16], presence of vascular invasion and perineural invasion (PNI) [17], poor tumor differentiation (G3) [18], tumor thickness or depth of invasion (DOI) [19], and neutrophil/lymphocyte ratio (NLR) [16]. Therefore, the AJCC TNM 8th edition has included new clinicopathological factors to improve LNM risk prediction: Tumor budding, the invasive front grading system, and the immunologic tumor microenvironment [20-22]. Additionally, the impact on HNSCC of non-tumor-related factors, such as medications like metformin and statins, is increasingly being explored [23]. Although OSCC prognosis is complex, achieving optimal care requires early identification of at-risk patients to guide treatment decisions and ensure close follow-up for those who may benefit.

This retrospective cohort study aimed to investigate the occurrence and timing of metastasis following curative treatment in patients with primary OSCC, as well as to evaluate clinical risk factors associated with cancer spread. We hypothesized that metastases are rare in patients initially staged as cN0 and that metastatic tendency can be predicted based on clinical variables.

## Material and Methods

A retrospective cohort study of patients with primary OSCC was conducted at Helsinki University Hospital, Helsinki, Finland. Data of patients treated for OSCC treated between January 2016 and December 2020 were evaluated retrospectively. Patients were identified through the records of the multidisciplinary Head and Neck Tumor Board of the Helsinki University Hospital, and detailed clinical information was retrieved from electronic patient records.

Patients’ oncological treatment and follow-up were carried out in accordance with the national protocol by the Finnish Society for Head and Neck Oncology, which follows the treatment recommendations of the European Society for Medical Oncology.

*Inclusion and exclusion criteria*

Patients who received curative treatment for a primary c/pN0 OSCC were included in the study. Patients defined as metastasis-free were required to have at least 12 months of follow-up data available. Patients were excluded if they were diagnosed with another simultaneous malignancy, experienced a local recurrence, or developed a second primary OSCC during the follow-up period.

*Study variables*

The outcome variable was the occurrence of OSCC metastasis during follow-up. The occurrence and timing of metastasis were determined based on the first detected metastasis event, either LNM or distant metastasis (with or without LNM).

Predictor variables included age, gender, smoking, heavy alcohol use, combination of smoking and heavy alcohol use, and tumor-related variables such as tumor size and location, depth of invasion (DOI, pathologically determined) treatment modalities, and regular use of metformin and statin medications. 

Patients were categorized by smoking habits into two groups: Non-smokers (including never-smokers, occasional smokers, and former smokers who had ceased smoking ≥5 years prior) and smokers (current smoker and former smokers who had ceased smoking <5 years prior). Heavy alcohol use was defined as the consumption of seven or more doses of alcohol per week (one dose corresponding to 10-12 g of pure alcohol), based on evidence suggesting that an intake of 70 g per week or more increases the risk of cancer [24]. Tumor sites were categorized as oral tongue, buccal mucosa, gingiva, palate, and floor of the mouth, according to The 10th revision of the International Classification of Diseases (ICD) codes. Tumor size was defined by T-category (T1-T4), based on the TNM Staging of Lip and Oral Cavity cancers - AJCC 7th  [25] and 8th Editions [26,27], depending on which was valid at the time of diagnosis. 

*Statistical analysis*

Patient metastasis-free survival analysis was conducted using the Kaplan-Meier method, an univariate survival analysis, and differences between curves were evaluated with the log-rank test. The start of follow-up was defined as the end of primary treatment. Multivariate Cox proportional hazards regression analysis was performed to estimate hazard ratios (HRs) with 95% confidence intervals (CIs). The statistical significance of the overall model and individual covariates was assessed using the likelihood ratio test and the Wald test. The proportional hazards assumption was tested using Schoenfeld residuals. Multicollinearity among covariates was evaluated using variance inflation factors. Smoking status was excluded from the final model as it violated the proportional hazards assumption by correlating other factors such as alcohol. All statistical analyses were performed using R software (The R Project for Statistical Computing) version 4.3.1.

*Ethical approval*

The study was approved by the Internal Review Board of the Head and Neck Centre, Helsinki University Hospital, Helsinki, Finland (HUS/66/2018).

## Results

A total of 524 patient charts with primary OSCC were reviewed. After applying the exclusion criteria, 195 patients met the inclusion criteria and were included in the final analyses (Figure 1). Patient were aged from 26 to 101 years (mean=65.2 years) (Table 1), and comprised 107 males (54.9%) and 88 females (45.1%). Overall, 38 patients reported both smoking and heavy alcohol use (19.5%), 89 (45.6%) were smokers, and 44 (22.6%) had a history of heavy alcohol use. The site-distribution in descending order was as follows: Tongue (111 patients, 56.9%), gingiva (38 patients, 19.5%), floor of the mouth (31 patients, 15.9%), buccal mucosa (10 patients, 5.1%), and palate (5 patients, 2.5%). Altogether, 139 OSCC patients underwent some form of neck procedure (71.3%), and 31 (15.9%) received radiotherapy as adjuvant therapy. 

The mean and median follow-up times were 42 and 40 months (range 8-85 months). Of the 195 c/pN0 OSCC patients, 19 (9.7%) developed a metastasis during the follow-up (Table 2). LNM was the first detected metastasis in 15 patients, while DM was first detected in four. The mean time of metastasis occurrence was 13.2 months after surgery. Clinical symptoms or findings led to the detection of metastasis in 13 patients (68.4%). Of the 19 patients with metastasis, nine (47.4%) were diagnosed during routine follow-up visits: Three (15.8%) solely by routine radiological imaging and six (31.6%) by routine clinical examination. In the remaining 10 patients (52.6%), metastases were detected outside of routine follow-up: Seven (36.8%) during additional control visits due to clinical suspicion of metastasis, and three (15.8%) during examinations conducted for other reasons or unrelated conditions.

No statistically significant differences were observed between men and women in metastasis-free survival (Figure 2). T-class (*p=*0.015) was significantly associated with the occurrence of metastasis. In contrast, tumor anatomical site (*p=*0.29), neck procedure (*p=*0.43), and radiotherapy (*p=*0.5) were not statistically significant associated with metastatic disease (Figure 3). Non-smokers were more likely to develop metastases compared to smokers (*p=*0.03), and heavy alcohol use was also associated with metastasis occurrence (*p=*0.021).

No significant differences in metastasis-free survival were observed between users and non-users of statin (*p=*0.21) or metformin (*p=*0.69) medication (Figure 4).

In multivariate Cox regression analysis, heavy alcohol use (HR 4.05, 95% CI: 1.38-11.92, *p=*0.011) and depth of invasion (HR 1.10, 95% CI:1.01-1.20, *p=*0.021) were significantly associated with an increased risk of metastasis (Table 3). With a HR of 1.10, DOI increased the risk of metastasis by 10% per 1mm. Age, gender, site, neck procedure, radiotherapy, and T-class were not statistically significant predictors in this analysis.

**Discussion** 

The present study aimed to investigate the occurrence and timing of metastasis following curative treatment in patients with primary c/pN0 oral cavity squamous cell carcinoma. One in ten patients developed metastases during follow-up. Our hypothesis was partly confirmed, as heavy alcohol use emerged as an independent predictor of metastases. Consistent with previous findings [28], this study also identified DOI to independently increase the risk of metastasis. Surprisingly, gender, age, and tumor site were not significant for the metastases, nor were metformin or statin use associated with metastasis occurrence.

Heavy alcohol use was associated with an increased risk of metastasis, supporting the hypothesis that alcohol-induced OSCC may represent a distinct disease in terms of etiology. The frequent association between alcohol use and smoking may also contribute to the result. We previously reported that alcohol affects the cancer site in OSCC, and it has its own pathways in cancer development [29]. Secondly, studies focusing on laryngeal squamous cell carcinoma have discovered that the development of recurrence is linked to alcohol through alterations in laryngeal microbiota. Certain bacterial and fungal genera related to alcohol consumption were found to be independent risk factors for recurrence in laryngeal squamous cell carcinoma [30]. In contrast, smokers often present with more advanced and aggressive disease [31], which in this study might have led to their exclusion, as we only focused on non-metastatic diseases. Smoking status was excluded from the final multivariate regression model, however, as it violated the proportional hazards assumption, indicating that its effect on the risk of metastasis did not remain constant over time. Although a stratified Cox analysis could have been considered, smoking showed collinearity with other covariates in the model, limiting its interpretability.

Tumor subsite has also been studied in relation to the risk of regional recurrence. Previous studies have concluded that that the incidence of LNM is higher for tumors located in the inferior regions of the oral cavity, with the tongue showing the highest rates of LNM and occult LNM [17,32,33]. Tumors of the floor of the mouth also carry a higher risk of regional recurrence, even in early-stage tumors, due to the rich lymphatic network [34]. Conversely, the buccal mucosa, mucosal lip, hard palate, and alveolar ridge subsites have demonstrated lower rates of occult LNM [17,35]. Surprisingly, no significant differences in the risk of metastasis were found between the subsites in the current study. Unlike metastatic disease, tumor location in c/pN0 cases seems to have limited prognostic relevance. This suggests that the etiology of occult metastasis may be influenced more by intrinsic tumor biology than by specific anatomical origin.

Factors previously shown to associate with the development of LNM include primary tumor size and thickness [33], tumor differentiation, vascular invasion [36], perineural invasion, advanced tumor stage, desmoplasia [37], a high level of lactate hydrogenase and serum sodium, and various molecular pathways [38]. These variables should be examined in detail in further studies also in c/pN0 tumors. This study focused on clinical variables and treatment-related prognoses, with depth of invasion (DOI) being the only histopathological variable included in the analysis. On the other hand, it is likely that individual factors may not explain the tendency for poor prognosis.

Furthermore, three patients without LNM developed DM. While the presence of LNM is known to increase the risk of DM, and cN2-3 patients are more susceptible to develop DM [39,40], some patients develop DM without prior cervical LNM, similar to those in the present study [41]. The presence of micrometastases in lymph nodes may explain this phenomenon [41]. Further research is needed to better understand the predisposing factors that lead to distant spread in the absence of cervical LNM, as DM is associated with a significant decline in prognosis [14].

Medications that could potentially decrease the risk of LNM were briefly examined in this study. Metformin has been suggested as a potential adjunctive agent in HNSCC, with evidence suggesting that it may improve therapeutically through its anticancer effects [23]. It has been reported to act against cancer initiation and progression by enhancing the radiotherapy-sensitivity of tumors [42]. Likewise, statins have been found to have anticancer effects by inhibiting cell proliferation through negative regulation of the cell cycle [43]. The exact pharmacological mechanisms by which statins influence the regulation of cancer cells remain unclear, however, several molecular mechanisms have been suggested, such as cell cycle arrest, apoptosis, and autophagy [43-46]. In the present study, neither statins nor metformin showed an association with LNM among OSCC patients, but, subgroup sizes were small for definitive conclusions. It remains possible that these drugs may influence specific subtypes of OSCC. 

Most patients in this study with metastases experienced symptoms related to the spread of the disease (78.9%), and in 68.4%, clinical symptoms or findings led to the diagnosis (Table 2). Still, the National Comprehensive Cancer Network (NCCN) guidelines (1.2015) provide no specific recommendations regarding the modalities for detecting cervical LNM, besides the length of follow-up period. Furthermore, most recurrences are self-reported by the patients themselves [47]. In this study, the number of metastases detected through patient-initiated contact equaled those identified during routine follow-up visits. Palpation, computed tomography (CT), magnetic resonance imaging (MRI), ultrasonography (US), and positron emission tomography (PET) are currently used during follow-up, with most of the metastases discovered by palpation [48,49]. The accuracy of palpation is influenced by several factors, however, such as the patient’s body mass index (BMI) and the diameter of the tumor [50]. In contrast, patients with DM often present with general health-related symptoms and are therefore more likely to be diagnosed through radiological examination. As such, the importance of routine clinical evaluation and imaging should be re-evaluated.

This study has certain limitations. First, the sample size was relatively limited (n = 195), potentially increasing the risk of random variation and ultimately influencing the observed associations. However, the data were collected from the largest hospital treating OSCCs in Finland over a five-year period. The second limitation is that the treatment modalities and scientific evidence develop rapidly, and not all information affecting the prognosis has been considered in the retrospective data. Third, histopathological data were limited, particularly for features such as perineural and vascular invasion, known to influence the risk of LNM, which restricts the ability to comprehensively model metastatic risk. Moreover, histopathological limitations include the merging of AJCC 7th and 8th edition staging systems, which could affect the results since the latter incorporates DOI and extranodal extension (ENE) to the staging to improve prognostic accuracy. This leads to upstaging. Finally, although patients were followed for at least one year, late-occurring metastases may not have been captured.

## Conclusions

Since metastases can arise even long after the initial diagnosis, it is important to ensure a sufficiently long follow-up period of up to four years with both radiological and clinical examinations. In addition, patients should be advised to contact their healthcare provider if they notice changes in their general condition or new clinical findings in the neck. Overall, before considering a more extensive follow-up period for c/pN0 patients, it is essential to determine which patients are most likely to benefit. Particular attention should be given to patients with heavy alcohol use, and the importance of DOI in assessing the risk of metastasis should be acknowledged. Further research is needed to identify markers that can help prioritize healthcare resources for those at the highest risk of disease recurrence.

## Figures and Tables

**Table 1:** Table 1: Descriptive statistics of 195 patients with oral squamous cell carcinoma.

**Patient characteristics**	**n**	**%**
Age (Years)		
*Range*	26-101	
*Mean*	65	
*Median*	67	
Gender		
*Male*	107	54.9
*Female*	88	45.1
Smoking and heavy alcohol use		
*Yes*	38	19.5
*No*	157	78.9
Smoking*		
*Yes*	89	45.6
*No*	106	54.4
Heavy alcohol consumption**		
*Yes*	44	22.6
*No*	151	77.4
Site		
*Tongue*	111	56.9
*Gingiva*	38	19.5
*Floor of the mouth*	31	15.9
*Buccal mucosa*	10	5.1
*Palate*	5	2.5
T-Classification		
*T1*	112	57.4
*T2*	48	24.6
*T3*	12	6.2
*T4*	23	11.8
Primary neck procedure		
*Any*	139	71.3
*Neck dissection*	99	50.8
*Sentinel lymph node removal*	40	20.5
*No*	56	28.7
Radiotherapy		
*Yes*	31	15.9
*No*	164	84.1
Metformin medication		
*Yes*	26	13.3
*No*	169	86.7
Statin medication		
*Yes*	40	20.5
*No*	155	79.5
Any metastasis		
*Yes*	19	9.7
*No*	176	90.3

* Current smoking or cessation less than 5 years ago. **7 alcohol portions per week or more.

**Table 2:** Table 2: Characteristics of the 19 patients with metastasis during follow-up of 195 oral squamous cell patients.

	**n**		**% of 195**
**Any**	19		9.7
Lymph node metastasis*	15		7.7
Distant metastasis*	4		2.5
**Detection of metastasis**		**% of 19**	
*Routine control visit*	9	47.4	4.6
*Solely by radiological imaging*	3	15.8	
*Routine clinical examination*	6	31.6	
*Other examination*	10	52.6	5.1
*Additional control visit due to suspicion of metastasis*	7	36.8	
*Examination due to other reason or disease*	3	15.8	
**Clinical symptoms or findings led to the detection of metastasis**			
*Yes*	13	68.4	6.7
**Types of all clinical symptoms or findings of metastasis**			
**Any**	15	78.9	7.7
*Palpable or otherwise detectable resistance*	13	68.4	
*Pain*	2	10.5	
*Infection*	1	5.3	
*Dysarthria and dysphagia*	1	5.3	
Other**	1	5.3	

*first detected metastasis ** tiredness, shortness of breath, chest pain, fever, decline in general condition

**Table 3:** Table 3: Multivariate Cox regression analysis of risk factors for metastasis in patients with OSCC.

	**Multivariate Cox regression analysis**		
Variable	**HR**	**95% CI**	*P-* **value**
Age	0.99	0.95-1.04	0.811
Female (ref. male)	2.29	0.72-7.31	0.162
Alcohol consumption 7 portions/week	4.05	1.38-11.92	0.011
Tongue (n=111)	reference		
Gingiva (ref. tongue)	0.59	0.10-3.72	0.578
Floor of the mouth (ref. tongue)	0.21	0.02-1.93	0.167
Palatinum (ref. tongue)	2.81	0.26-30.71	0.398
Buccal mucosa (ref. tongue)	0.78	0.09-6.55	0.818
Depth of invasion	1.10	1.01-1.20	0.021
No neck treatment (n=56)	reference		
Neck dissection (ref. no treatment)	1.64	0.36-7.41	0.521
Sentinel node biopsy (ref. no treatment)	0.76	0.13-4.43	0.756
No radiotherapy (n=164)	reference		
Radiotherapy (ref. no treatment)	0.36	0.07-1.81	0.214
T1 (n=112)	reference		
T2 (ref. T1)	0.72	0.15-3.39	0.672
T3 (ref. T1)	0	0-inf	0.998
T4 (ref. T1)	0.95	0.10-9.27	0.965

HR: Hazard ratio, CI: Confidence interval.

**Figure 1:**
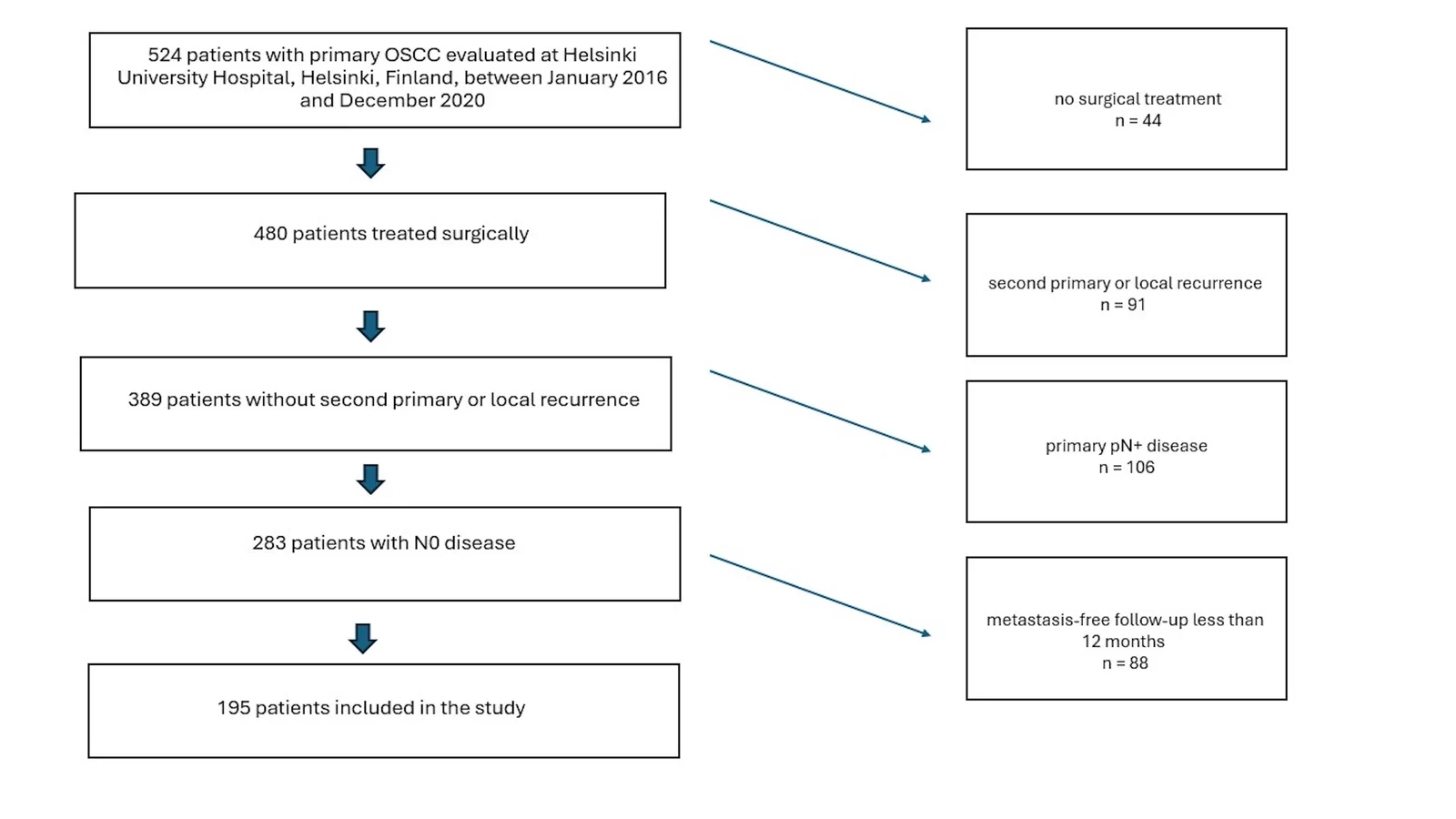
Fig. 1: Flowchart of patients included in the study.

**Figure 2:**
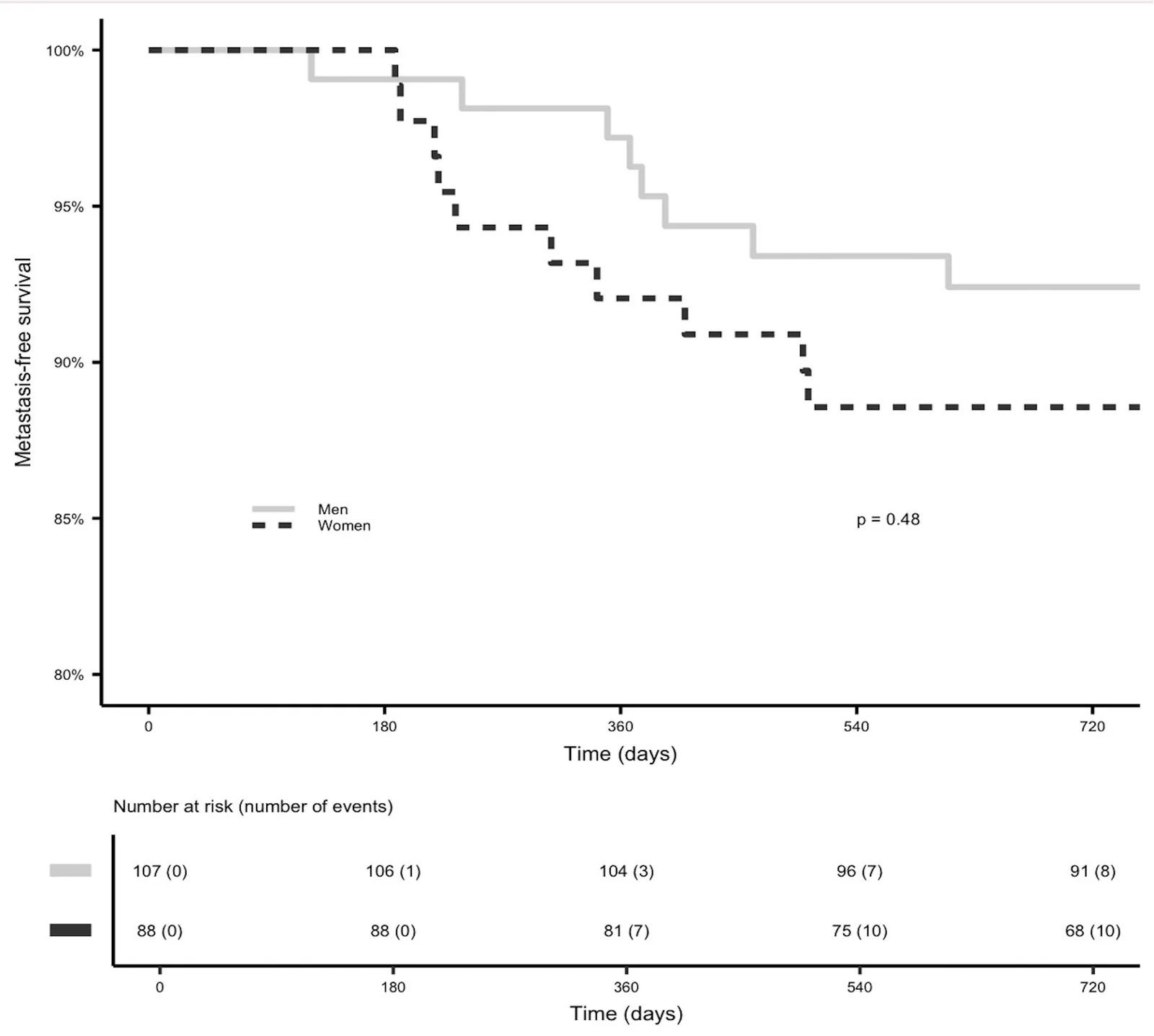
Fig. 2: Kaplan-Meier curves of metastasis-free survival in c/pN0 by gender. The difference between genders was not statistically significant.

**Figure 3:**
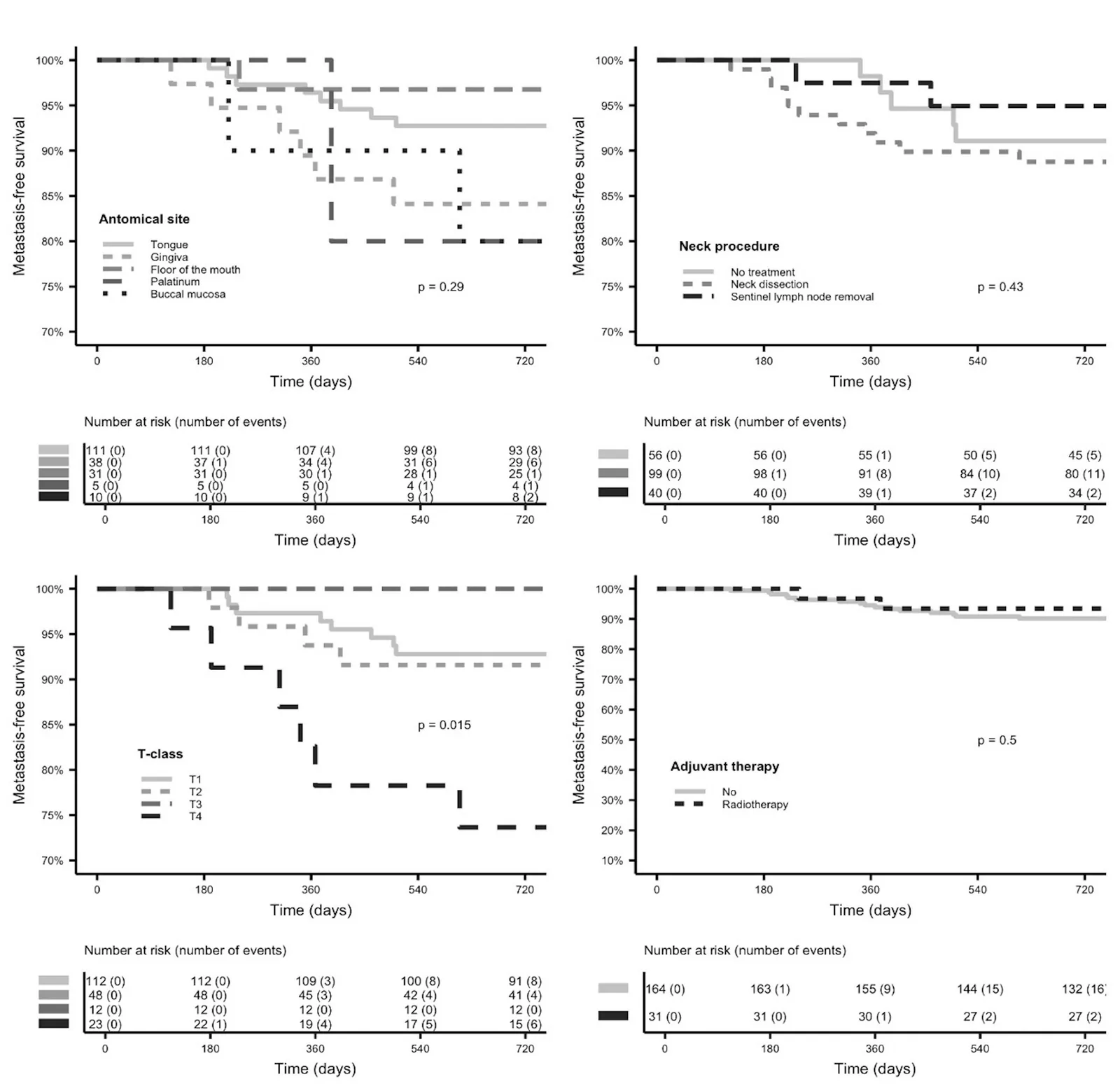
Fig. 3: Kaplan-Meier curves of metastasis-free survival in c/pn0 patients by tumor anatomical site, T classification, neck procedure and adjuvant therapy. Only T-class was significantly associated with the occurrence of metastasis (p=0.015).

**Figure 4:**
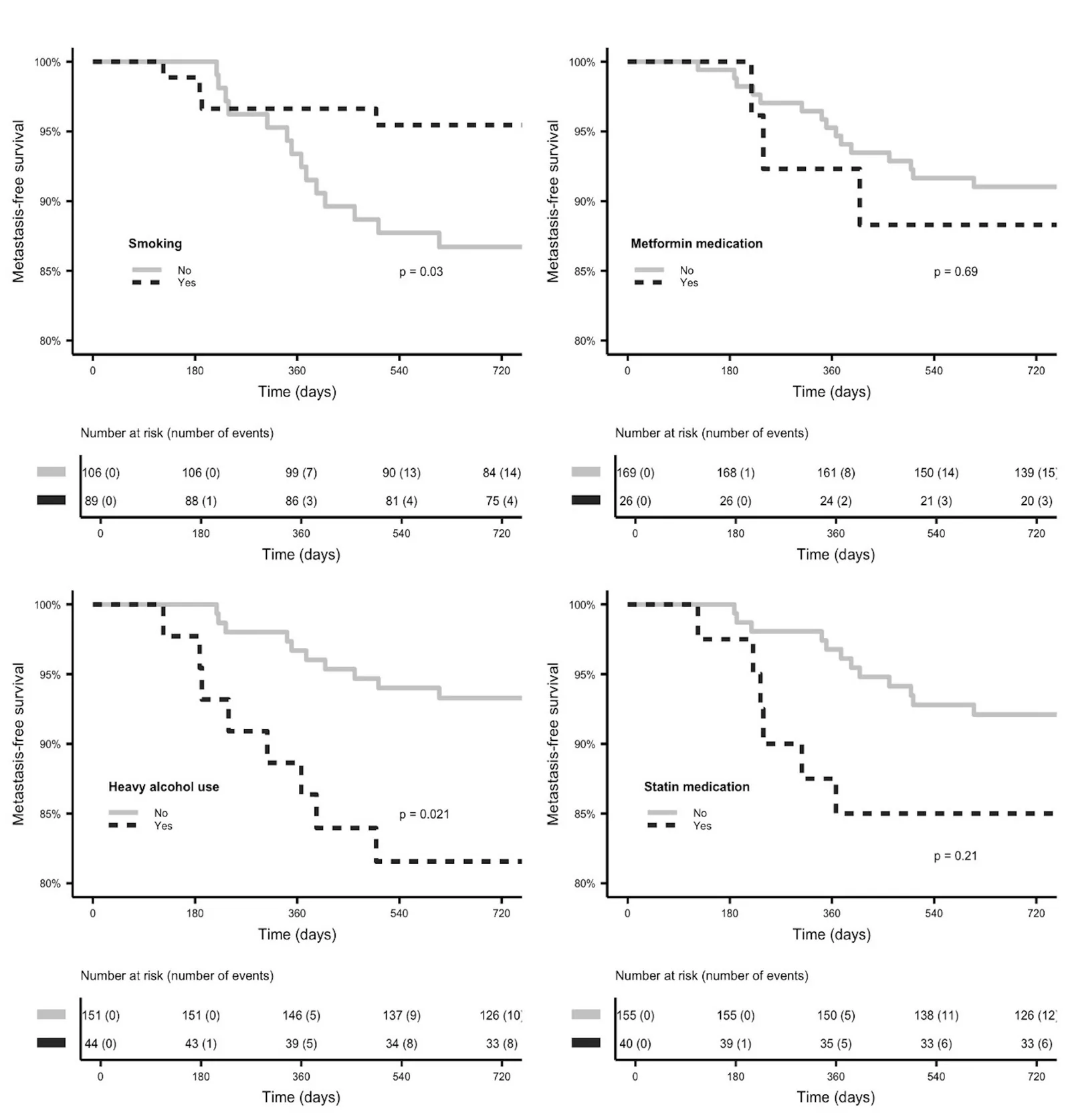
Fig. 4: Kaplan-Meier curves of metastasis-free survival in c/pN0 patients by smoking status, metformin use, heavy alcohol use, and statin medication. Non-smokers were more likely to develop metastases compared to smokers (p=0.03), and heavy alcohol use was also associated with metastasis occurrence (p=0.021). Statin and metformin use were not associated with the occurrence of metastases.

## Data Availability

Declared none.
